# Actualities on molecular pathogenesis and repairing processes of cerebral damage in perinatal hypoxic-ischemic encephalopathy

**DOI:** 10.1186/1824-7288-36-63

**Published:** 2010-09-16

**Authors:** Giuseppe Distefano, Andrea D Praticò

**Affiliations:** 1 Department of Pediatrics, Division of Neonatology, University of Catania, Catania, Italy

## Abstract

Hypoxic-ischemic encephalopathy (HIE) is the most important cause of cerebral damage and long-term neurological sequelae in the perinatal period both in term and preterm infant.

Hypoxic-ischemic (H-I) injuries develop in two phases: the ischemic phase, dominated by necrotic processes, and the reperfusion phase, dominated by apoptotic processes extending beyond ischemic areas. Due to selective ischemic vulnerability, cerebral damage affects gray matter in term newborns and white matter in preterm newborns with the typical neuropathological aspects of laminar cortical necrosis in the former and periventricular leukomalacia in the latter.

This article summarises the principal physiopathological and biochemical processes leading to necrosis and/or apoptosis of neuronal and glial cells and reports recent insights into some endogenous and exogenous cellular and molecular mechanisms aimed at repairing H-I cerebral damage.

## Introduction

Hypoxic-ischemic encephalopathy (HIE) is the most important cause of cerebral damage and long-term neurological sequelae in the perinatal period both in term and preterm infant [1].

Until few years ago, the main acquisitions on the physiopathogenetic mechanisms of this affection came from experimental studies on animals. Recently, the progress in brain immunocytochemistry, the discovery of specific neuropathologic biochemical markers and, above all, the development of new and sophisticated techniques of nuclear magnetic resonance (NMR), i.e. diffusion weighted imaging (DWI), diffusion tensor immaging (DTI), tractography (Fibre-Tracking Techniques) and magnetic resonance spectroscopy (MRS), have collected data directly from human infants [2].

## Physiopathology

Besides the main role of perinatal asphyxia, a key factor in the genesis of HIE is the loss of "cerebral blood flow (CBF) autoregulation", a protective mechanism that maintains stable cerebral blood flow velocity (CBFV) in normal infants, regardless of variations of systemic arterial pressure. This mechanism is expressed by reflex modifications of cerebral arteriolar tone induced by secretion of some humoral factors with vasoconstrictive or vasodilatative action. In case of rising pressure, the release of vasoconstrictors (such as endothelin and thromboxane) that increases arteriolar resistances, reduces CBFV, while in the event of low pressure the release of vasodilators (such as prostacicline and nitric oxide) reduces arterioral resistances and increases CBFV [3]. It has to be underlined that these autoregulator mechanisms are functionally immature in preterm infants and can be jeopardised by perinatal asphyxia due to vasoparalysis induced by increased PaCO2 and acidosis [4].

In presence of impaired autoregulation, CBF becomes passive to pressure stimuli. In this way, rising pressure increases CBF and can cause haemorrhagic phenomena, while low pressure reduces CBF and can cause ischemic phenomena. It has to be kept in mind that systemic arterial hypotension is frequent in high-degree premature infants who present cardiac contractile function and sympathetic vascular tone immaturity, and in all newborns with perinatal asphyxia, resulting from impaired myocardial contractility induced by hypoxia and acidosis [5]. This explains why these infants are particularly exposed to cerebral ischemia.

Ischemia reduces CBF and thus supplies less oxygen and fewer nutrients (especially glucose, fundamental for brain energetic metabolism) and this in turn induces neural and glial cells distress triggering off cerebral damage. This is prevalently necrotic damage in cases of severe asphyxia and apoptotic in moderate asphyxia [6]. The severity and extension of brain damage are strictly related to intensity, timing and duration of hypoxic-ischemic (H-I) insult. The more intense and long-lasting it is, the greater is the number of neuronal and glial cells which die.

## Mechanisms of cerebral injury

Numerous studies show that H-I cerebral damage develops in two phases: the first or "ischemic phase" dominated by necrotic processes in the ischemic areas and the second or "reperfusion phase" dominated by apoptotic processes extending beyond ischemic areas. This second phase takes place two - six hours after H-I insult, such latency constituting a useful window in which therapeutic measures can be able to stop the evolution of cerebral damage [6,7].

### Ischemic phase

**Figure 1:**
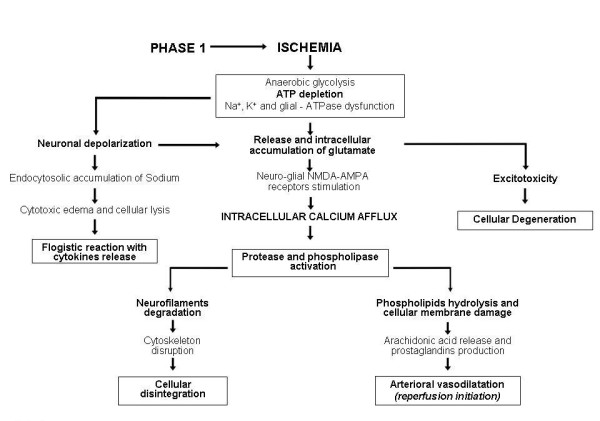
**Pathogenesis of hypoxic-ischemic cerebral damage: potential sequences of events developing during ischemic phase (Phase 1)**.

In this phase, the crucial event triggering a cascade of chain reactions is represented by ATP depletion secondary to anaerobic glycolysis and metabolic acidosis induced by hypoxia [8]. Reduced ATP availability determines the dysfunction of ATPase systems, in particular Na^+^, K^+^-ATPase and glial-ATPase. Na^+^, K^+^-ATPase dysfunction determines depolarization of neurons causing intracellular sodium and water accumulation with cytotoxic edema and/or cell lysis followed by inflammatory reaction with cytokines release. At the same time, neuronal depolarization induces delivery of glutamate (a neuroexcitatory aminoacid) which tends to accumulate in the intersynaptic and intercellular spaces because of the dysfunction of glial-ATPase, an astrocytic enzyme normally delegated to its reuptake. Glutamate stimulates specific NMDA and AMPA neuro-glial receptors and hence determines a massive intracellular entry of calcium that activates some endocellular enzymes including protease and phospholipase. Protease degrades neurofilaments and can determine cytoskeleton rupture with disintegration of the cellular body; phospholipase hydrolyzes phospholipids and can damage cellular membrane and induce the release of arachidonic acid with consequent production of vasodilator prostaglandins that give rise to reperfusion of ischemia (Figure 1). Furthermore, excessive amounts of glutamate can cause excitotoxicity leading to the death of neurons and glial cells [8].

### Reperfusion phase

**Figure 2:**
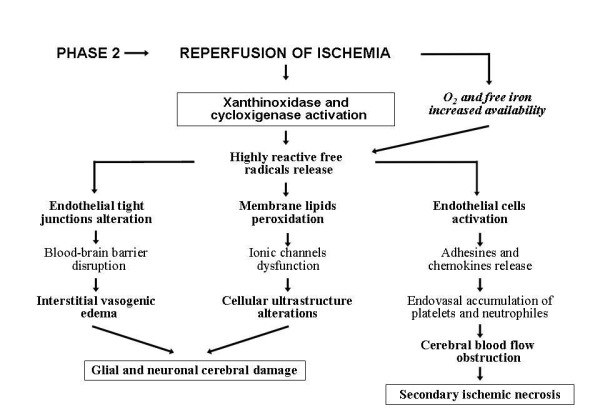
**Pathogenesis of hypoxic-ischemic cerebral damage: potential sequences of events developing during ischemia reperfusion (Phase 2)**.

In this phase, recovery of ischemia increases O_2 _availability and hence activates xantine-oxidase and ciclo-oxygenase enzymes and generates reactive oxygen species (ROS) responsible for oxidative cellular damage [9]. The formation of free-radicals, some being highly reactive such as hydroxyl radical, in addition to oxygen is also supported by a major haematic concentration of free iron due to hypoxia-induced acidosis that is known to detach the iron bonded in circle to transferrine [10]. These oxidative processes, in fact, can be blocked by deferoxamine, an iron-chelator that experimental studies in lambs have shown capable of preserving cortical cells membrane stability and electrocortical brain activity when used during early reperfusion after H-I insult [11]. Free radicals determine various harmful reactions. They can induce deep alterations in cellular ultrastructure through membrane lipids peroxidation and ionic channels dysfunction. At same time, by damaging endothelial tight-junctions, they disrupt the blood-brain barrier (BBB) integrity causing an interstitial-vasogenic edema which worsens cerebral damage. Another dangerous biochemical action of free radicals is the activation of endothelial cells that induce adhesins and chemokines releases and thus can determine endoarteriolar accumulation of neutrophiles and platelets with alteration of CBF leading to secondary ischemic necrosis (Figure 2). Other biochemical injuries caused by free radicals are those connected to the development of apoptosis. This can be due to activation of caspases enzymatic system resulting from cytochrome C release secondary to oxidative mitochondrial damage, and to induction of proapoptotic genes, i.e. Bax-gene [12]. Studies on the cortical tissue of rat pups exposed to 100% O_2 _after neonatal H-I insult have shown that hyperoxia determines an early increase in intranuclear and total nuclear Bax protein levels followed by subsequent Bax redistribution to the mitochondria and endoplasmic reticulum with activation of biochemical signals inducing apoptosis and inflammation [13]. Oxygen concentration during resuscitation must be reduced to prevent these dangerous effects. During cerebral ischemia reperfusion, increased production of nitric oxide, due to neural and endothelial nitric oxide-synthetase activation, can cause further damages. In the first case, the intraparenchimal release of nitric oxide interacts with hydroxyl radical and leads to the formation of peroxynitrite, a highly cytotoxic agent for cerebral tissues. In the second case, endovasal release of nitric oxide causes vasodilatation with increased endothelial permeability and facilitates intracerebral diffusion of cytokines and reactive molecules that contribute to the development of neuronal and glial cells damage [14].

## Location of injury in relation to gestational age

The location of cerebral damage differs depending on gestational age and involves primarily gray matter in term infants and white matter in prematures. This selective vulnerability of different cellular populations is related to maturational events connected to cerebral vascular system development. The neonatal encephalic regions that are more exposed to the risk of ischemia are those localized in the border zones between the end-fields of the major cerebral arteries (anterior, middle and posterior), where normal perfusion rate is basically low for the absence of anastomotic connections [15]. These border zones, in fact, according to watershed concept (Volpe JJ, 2008) are the brain areas most susceptible to a fall in cerebral perfusion pressure. The watershed concept, based on the analogy with an irrigation system supplying a series of fields with water, emphasizes the vulnerability of the "last fields" when the head of pressure falls and have received ample experimental support in several developing animal models of hypoxic-ischemic cerebral damage [16].

In term infants, hypoperfused areas are localized superficially in the parasagittal cerebral regions, with ischemic injuries interesting cortical gray matter and adjacent layers of subcortical white matter. The characteristic neuropathologic aspects are represented by cortical laminar necrosis with subcortical leukomalacia.

In preterm infants, hypoperfused areas involve periventricular white matter regions. Therefore, in these subjects the classical neuropathologic aspect is that of periventricular leukomalacia (PVL), where damaged cells are made up of immature oligodendrocytes or premyelinating oligodendrocytes (pre-OLs) that ensheath axons in preparation for full differentiation to myelin-producing oligodendrocytes [17]. In preterm newborns, in addition to the peculiarity of periventricular arterial vascularisation, PVL is also favoured by other factors related to prematurity such as metabolic hyperactivity of periventricular encephalic areas - where intense processes of proliferation, differentiation and migration of glial and neuronal cells occur, raising oxygen and nutrients demand - and, above all, the particular vulnerability of immature oligodendrocytes to the oxidative stress due to their poor content of anti-oxidizing enzymes [18,19]. Another important role is played by the lack of neuroprotective factors such as neurotrophines and oligotrophines that are trophic substances capable of supporting brain development and inhibiting apoptotic phenomena. In the first phases of pregnancy, these factors are produced by the mother and reach the fetus through the placenta, whereas at the end of the pregnancy they are synthesized by the same fetus. Availability of such factors is therefore strongly deficient in preterm infants, especially in highly premature ones [16,20,21].

## Neuropathology of PVL

From a pathological point of view, PVL can be distinguished in two forms: focal and diffuse. The focal form is less frequent and related to severe H-I insults. This form involves deep layers of white matter surrounding long penetrating arteries terminations and is characterized by macro or microscopic necrotic foci - where destruction of all cellular elements and axonal distruption occur - respectively evolving, over several weeks, to multiple cysts (easily visible to cranial ultrasonography) and to glial scars [22]. The neuropathological sequelae of focal necrotic lesions are correlated with cerebral palsy (spastic diplegia) that affects 5-10% of very low birth weight infants with PVL [18].

**Figure 3:**
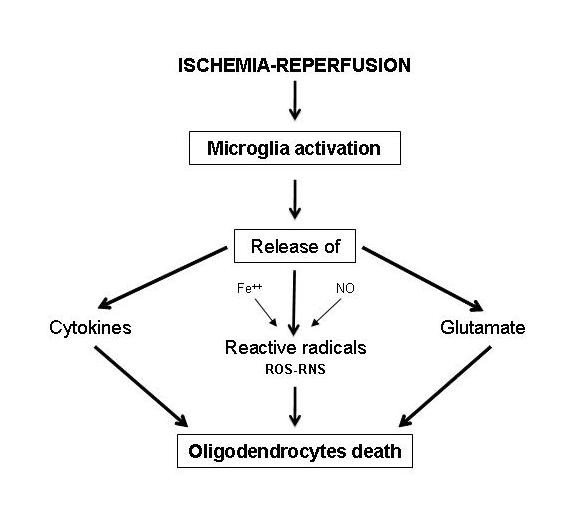
**Pathogenesis of periventricular leukomalacia**. Role of microglia activation.

**Figure 4:**
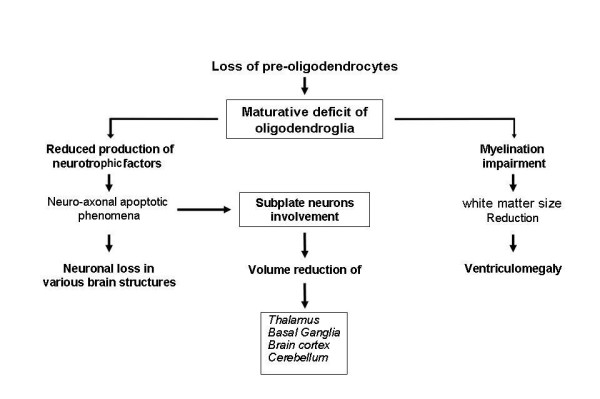
**Consequences of pre-oligodendrocytes loss in diffuse PVL**.

The most frequent diffuse form is related to moderate H-I insult and occurs in central white matter areas supplied by terminations of penetrating short arteries. It consists of diffuse processes of astrogliosis and microgliosis associated to pre-OLs loss [23,24]. This later form is undistinguishable from cranial ultrasonography, appearing as a not specific diffuse periventricular hyperechogenicity that can be identified by DW and DT MRI [25] and it is clinically correlated to cognitive behavioral deficits affecting 25-50% of PVL cases [18]. In the pathogenesis of diffuse PVL an important role is played by the activation of microglia, a cerebral supporting structure constituted by immunocells with macrophagic function present in large numbers in the developing periventricular white matter [26] (Figure 3). Such activation is associated with the release of nitric oxide, free radicals, glutamate and cytokines, substances able to induce pre-OLs death. Because of the lack of anti-oxidizing defences and the exuberant expression of glutamatergic and cytokinic receptors, these cells are particularly sensible to oxidative, excytotoxic and inflammatory injuries [27,28]. The susceptibility of these cellular elements to oxidative stress is also increased by their high content of free iron, a molecule indispensable for differentiation in mature oligodendrocytes, and by the presence of nitric oxide, that favour the formation of extremely reactive oxygen and nitrogen radicals. In diffuse PVL, pre-OLs damage causes a maturative deficit of oligodendroglia with subsequent dysfunction or loss of actively myelinating OLs impairing nervous fibres myelination and reducing cerebral white matter with appearance of ventriculomegaly (Figure 4).

## Axonal disease in PVL

As white matter contains both oligodendroglial and axonal components it is still not clear whether, in diffuse PVL, H-I injuries involve only the oligodendrocytes or also the axons. While axonal damage has long been recognized as a classic feature of focal necrotic lesions [29,30], observations of axonal injuries are only fragmentary in the diffuse form of PVL [31]. Moreover, in a recent study on fractin, a biochemical marker of apoptosis, Haynes et al. demonstrated the presence of widespread axonal damage in non-necrotic PVL contributing to reduced white matter volume revealed by volumetric MRI in long term survivors [32]. The cause of these axonal alterations is not yet clear, but it is probably secondary to oligodendroglial injuries. Myelinating oligodendrocytes play a critical trophic role in axonal development, survival and function, given the important effects of myelin-related proteins and OL-specific signals in long-term viability, thickness and conduction of axons [33,34]. Therefore by compromising neurotrophic factors release, pre-OLs loss can induce failure of axonal development and/or axonal degeneration that involving, through retrograde and anterograde trans-synaptic effects, cortical-thalamic projection, commissural and association fibres lead to volume reduction of cerebral cortex and thalamus [35]. This is mainly due to the fact that axonal alterations affect "subplate neurons", a transient population of neurons that are abundant in cerebral white matter, reach a maximum during the peak period for the occurrence of PVL (24-32 weeks of gestation) and play a crucial role in the development of the thalamus and brain cortex [36-38] (Figure 4). Volumetric MRI analyses of preterm infants with diffuse PVL, at term-equivalent age or older, have shown that, besides cortex and thalamus, volume reduction can involve other neuronal structures such as basal ganglia, cerebellum and brain stem [39,40]. Recent neuropathological studies showed that volumetric reduction of these structures was consistent with the finding of neuronal loss and gliotic processes [22,41]. These observations postulate the presence of a more complex "neuronal/axonal disease" accompanying oligodendroglial lesions in diffuse PVL. Therefore, the previously reported widespread axonopathy observed in non-necrotic PVL could be, to some extent, expression of degenerative processes secondary to primitive death of neuronal cell bodies in the gray matter of cortical and subcortical structures (especially the thalamus whose axons project to and from the cerebral cortex). Axonal impairment is suggested by NMR studies utilizing DTI which, in various fibre tracts of premature infants with non cystic PVL, as early as term equivalent age, showed a diminished relative anisotropy, an MRI measure of preferred directionality of diffusion [42,43].

**Figure 5:**
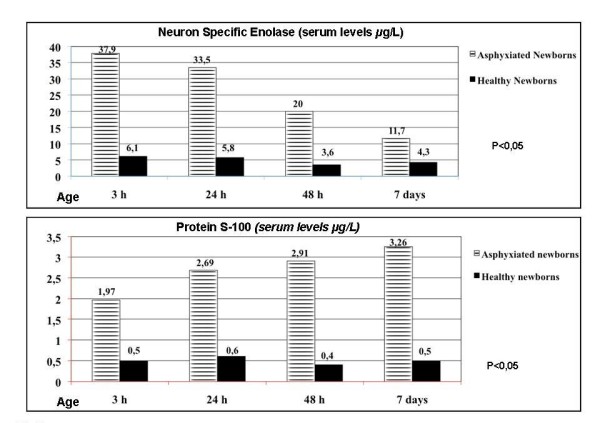
**Neuron specific enolase (NSE) and protein S-100 (PS-100) mean serum levels at 3, 24, 48 hours and 7 days of life in preterm infants with severe perinatal asphyxia**.

As reported in several studies in literature, brain damage in HIE can be detected by serum dosage of some biochemical markers of cerebral hypoxia, such as adrenomedullin, protein S-100B, Activin A [44-48]. Recently, the coexistence of neuro-axonal and glial alterations in PVL have been documented by our group studying serial serum levels of neuron-specific enolase (NSE) and protein S-100 (PS-100) in the first week of life, as biochemical markers of brain hypoxic injuries involving respectively gray and white matter. In thirty premature infants with perinatal asphyxia (severe in 15 cases and moderate in 15 cases) and neurosonographic findings suggesting diffuse white matter injury (periventricular echodensities/echolucences) or cystic PVL, NSE and PS-100 mean serum levels, at 3,24,48 hours and 7 days of life, were significantly elevated at all the time intervals compared to control group, the highest values being observed in the group of neonates with severe asphyxia. In these severely asphyxiated neonates, NSE values decreased constantly from birth to the seventh day of life, while PS-100 increased progressively over the 7 days [49] (Figure 5).

## Neurogenetic and gliogenetic processes after H-I injury

**Figure 6:**
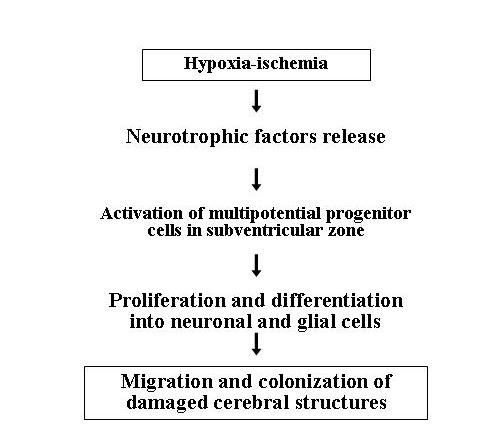
**Effects of hypoxic stimuli on cerebral subventricular zone resident progenitor cells**.

Recent studies in brains damaged by H-I insult have demonstrated that some cellular mechanisms can be activated in order to repair cerebral injuries [17]. These mechanisms involve the neural stem/progenitor cells (NSP_S_) normally resident in the subventricular zone (SVZ) of the mammalian brain that can be stimulated by hypoxia-ischemia to proliferate and differentiate into both neurons and oligodendrocytes. Yang et al have observed that H-I insult expanded the regenerative capacity of NSPs in the SVZ of the injured hemisphere in a neonatal rat model of H-I cerebral injury produced by unilateral common carotid artery ligation [50]. This robust proliferative response of SVZ-progenitors was accompanied by their capacity to differentiate into neuronal and glial elements, to migrate from the ipsilateral SVZ and to colonize the damaged structures (Figure 6). These observations raise the interesting possibility that these new cells may play an important role in repairing neuronal and glial losses related to HIE. Therefore, NSP_S _in SVZ could be a valuable target for therapeutic strategies to enhance recovery after cerebral H-I injury.

In this field of researches related to the possibility of repairing damaged cerebral structures, studies utilizing some pluripotent cells discovered in the stroma of adipose tissue of mice, rats, non human primates and humans are intriguing. These adipose stromal cells (ASC) can exhibit differentiation into neural and glial elements in vivo and in vitro and are capable of secreting potent neurotrophic factors [51]. In a rat middle cerebral artery occlusion model of ischemic brain injury, Kang et al [52] observed that intracerebral transplantation of human ASC was followed by migration of these cells to areas of ischemic damage and by expression of neuronal specific markers in conjunction with functional benefit. Therefore, therapeutic ASC could be an opportunity for developing treatments that will reverse or prevent the effects of H-I injury. Their clinical use, however, is strongly limited by the existence of the blood-brain barrier (BBB) that makes the human brain refractory to targeting of cell-sized agents delivered through the peripheral system. As intracerebral transplantation for bypassing BBB is a very invasive delivery method that cannot be proposed for human newborns, recently Wei et al. have designed a study to evaluate whether the same beneficial effects against H-I brain damage could be obtained with the neurotrophic factors secreted by ASC during culture, delivered through the peripheral venous system both preceding and following H-I injury [53]. Their results, obtained in a rat model of H-I injury, showed that infusion (1 hour before or 24 hours after injury induction) of concentrated medium (CM) from cultured ASC (ASC-CM) significantly protected against the hippocampal and cortical volume loss observed in controls. Moreover, analysis of parallel groups for behavioural and learning changes at 2 months post-ischemia demonstrated that rats treated with ASC-CM performed significantly better than controls in Morris water maze functional tests commonly used for studying spatial learning in the rat [54]. These positive effects of ASC-CM are not surprising because the cultural milieu of ASC was rich in neurotrophic factors, particularly insulin like growth factor-1 (IGF-1) and brain derived neurotrophic factor (BDNF) which, respectively, protect against cerebral cells apoptosis and glutamate-excitotoxicity [55-57]. In non published data, the same authors [53] found that equal neuroprotective activity in vitro was exhibited by ASC-CM derived from human ASC, this suggesting its potential and positive utilization in preventing or attenuating neonatal HIE. In addition to beneficial effects connected to antiapoptotic mechanisms, some factors produced by ASC may be involved in the recovery of damaged tissues stimulating migration, homing and differentiation of brain progenitor cells resident in SVZ. This approach could be an interesting way to stimulate endogenous repair without the need for targeting donor cells to the brain. Such hypothesis is supported by the recent discovery of some substances, i.e. 1alpha/CXC chemokine and nerve growth factor able to stimulate proliferation, homing and differentiation of resident neural progenitor cells in adults with cerebral injuries[58,59].

If all these observations are confirmed by further systematic studies in the coming years, it is possible to speculate that, intravenous delivery of the milieu of factors secreted by ASC at 24-72 hours after H-I injury may represent a new promising therapeutic strategy for treatment of human neonatal HIE in addition to hypothermia that, currently, represents the most efficacious option for preventing or attenuating cerebral damage [60].

## Competing interests

The authors declare that they have no competing interests.

## Authors' contributions

GD is principal author. ADP carried out the paragraphs "Mechanisms of cerebral injury" and "Neuropathology of PVL". Both authors read and approved the final manuscript.
